# The Electrophysiology Lab of the Future

**DOI:** 10.19102/icrm.2025.16081

**Published:** 2025-08-15

**Authors:** John Cochran, Michael M. Shehata

**Affiliations:** 1 Department of Cardiology, Smidt Heart Institute, Cedars-Sinai Medical Center, Los Angeles, CA, USA

**Keywords:** Ablation, cardiac arrhythmias, education, electrophysiology lab, interventional electrophysiology, radiation exposure, virtual reality

## Abstract

Electrophysiology (EP) labs are fundamental in cardiovascular medicine, especially for the diagnosis and treatment of cardiac arrhythmias. Nowadays, continuous advances in technology have led to significant improvements in the design and functioning of EP labs, including the development of more sensitive and accurate sensors and algorithms as well as three- and four-dimensional imaging and guidance systems. However, there are still significant challenges related to the reduction of radiation exposure, space constraints, and the integration and compatibility between the different EP systems. Future advances in technology will lead to equipment and space improvements as well as the addition of advanced communication and collaboration tools, speech-recognition software, and the development of standardized data formats and centralized cloud-based data storage systems. The EP lab of the future will present multifunctional configurations that will integrate all the advances in technology, optimize workflows, and potentiate collaboration while assuring patient data protection.

## Introduction

Cardiology is a highly dynamic medical field driven by continual technological advancements and evolving medical insights. Hein Wellens stated, “there is a golden rule in cardiology that, every five years, 50% of our knowledge is replaced by new information.”^1^ This feature is reflected in the continuous developments in the field of electrophysiology (EP), where significant technological advances have been made in the last 50 years. EP labs are pivotal facilities to diagnose and treat cardiac arrhythmias. If one would have to design an EP lab today, the high number of technical possibilities would present a challenge to define the most relevant aspects of the space. Planning on the future requirements for EP labs will be fundamental to develop technological enhancements leading to improvement in treatment options, patient outcomes, and global accessibility, while reducing staff burden, institutional overhead, and space constraints. Future challenges for the improvement of EP labs include the integration of existing equipment with innovative technologies while dealing with space constraints, as well as the design of end-user–friendly and globally accessible tools. This article explores recent advancements in technology, hardware, infrastructure, data integration, workflow efficiencies, and education, providing a roadmap for creating more flexible, efficient, and accessible EP services globally.

## Background

Nowadays, complex arrhythmias can be treated better than ever before, although there are still challenges such as disparate pieces of equipment (ie, various inputs into a central monitor) or the lack of communication between the different recording, mapping, ablation, and imaging systems. In addition, the electronic medical record cannot integrate unique data and images that originate from the EP recording and mapping systems, preventing the use of a centralized patient data repository.

The typical EP lab contains different systems with multiple data displays, including electrogram (EGM) recording, mapping, intracardiac echocardiography (ICE), and robotic navigation systems, as well as ablation consoles and telemedicine or broadcasting technologies. This infrastructure is not easily transferrable to other locations and becomes “cemented” into one location or room, leading to procedure and staff scheduling constraints.

Although these disadvantages may be increasing and limiting, there are many improvements on the horizon that will continue to change the way EP is practiced. Importantly, treating physicians and patient care teams must be vocal to drive those innovations.

## Artificial intelligence and machine learning

Imaging modalities using machine learning (ML) algorithms are being developed to allow real-time processing of two-dimensional (2D) images into 3D and 4D reconstructions in a matter of seconds, enhancing the efficiency and real-time geometry reconstruction of the entire heart or chamber of interest without the need of additional labor or time-consuming catheter-based mapping.

The ability to use head movements or voice commands for mapping system manipulation across platforms is also needed. These advancements may allow for more accurate catheter manipulations with barely visible movements to achieve different views and imaging, without the need for separate mapping staff to guide the systems, thereby improving procedural workflows.

This technology must be adaptable to not only receive and analyze data but also to integrate that information as workable data that speak to associated EP systems, patient image repositories, or databases and host multiple artificial intelligence (AI) applications.

Kabra et al.^2^ outlined the possibilities of analyzing data in innovative ways to help drive “superior predictions about the behavior of complex systems” and defined what a clinical AI decision framework may look like. Importantly, the authors noted that both AI and ML have already had a significant impact in clinical EP, such as in electrocardiography (ECG), wearables for detection and diagnosis, and prediction and management of arrhythmias. ML has been used for ion channel and action potential research, to evaluate the effects of drugs on cardiac electrical function, for computational research of atrial fibrillation (AF) spatial patterns, for the prediction of ablation outcomes by localizing ectopic beats from simulated body surface maps, and for clinical decision support.^3^ In addition, ML has been shown to detect AF drivers, and it has been suggested that it may improve the accuracy of AF driver detection for targeted ablation treatment in patients.^4^

## Hardware and infrastructure

Equipment and space improvements must follow the two pathways described below to inclusively support and advance centers that have current equipment or space constraints as well as new design and construction of EP labs that will have advanced infrastructure built in.

### Flexible room configuration

The future of EP labs will embrace flexible room configurations, allowing multiuse environments. Multifunctional rooms will allow EP labs to adapt to various procedural requirements, optimizing space and resource utilization. The increasing prevalence of ambulatory service centers indicates the need for efficient EP lab configurations equipped with state-of-the-art technology. However, in the case of EP, fully ambulatory surgery center environments may separate the patient from vital hospital services in the event of a life-threatening complication, such as a pericardial tamponade or endovascular injury requiring surgical repair.^5^ Rather, improvements in shared space by using combined technologies, shared control rooms, and data works will increase lab functionality and benefits.

Improved environmental design may also be beneficial in increasing staff satisfaction and the work environment. An example is the use of glass walls emerging as a new standard in Europe as they provide improved aesthetics and lighting and improved sterilization, and glass also has continued durability over some other materials. Additionally, flexible ceiling solutions with the use of modular ceilings may be useful. This application can allow for fixed ceiling equipment such as imaging systems, lights, and booms to be moved easily if or when room layout changes are required and eliminates future construction requirements.

### Design to reduce radiation exposure

The use of fluoroscopy to guide many electrophysiologic procedures is necessary but exposes everyone in the EP lab to the deterministic and stochastic risks of ionizing radiation and exposes EP lab staff to the orthopedic risk inherent to wearing heavy protective lead garments. For patients, the risk of additional cancer after a standard procedure is small and roughly quantifiable (estimated increase in 1 new cancer per 750 patients exposed to a standard fluoroscopic procedure).^6^ For physicians and lab staff, the risk is more difficult to quantify as fluoroscopic technology and shielding techniques have changed over the years to reduce doses; however, some estimate a “professional lifetime attributable to excess cancer risk on the order of magnitude of 1 in 100” for interventional cardiologists^7^ (data are less available for electrophysiologists but can be expected to be roughly similar).

It is imperative to decrease radiation exposure to mitigate the risks of malignancy as well as orthopedic issues from lead apron use. In addition to minimizing fluoroscopy through imaging techniques not involving radiation (ICE, mapping), shielding systems that allow the operator to operate with fluoroscopy but without wearing lead should be integrated into practice more thoroughly. Some of these require the use of ceiling boom-mounted systems that move with the operator or additional upfront setup at the start of the case to act as a barrier and eliminate the need to wear lead. Existing examples include the Rampart IC (RAMPART ic, LLC, Birmingham, AL, USA), EggNest (Egg Medical, Arden Hills, NM, USA), Lemer Cathpax (Lemer Pax, La Chapelle-sur-Erdre, France), and Zero-Gravity (Biotronik, Berlin, Germany) systems.

### Advanced imaging and mapping technologies

Imaging and mapping technologies are critical components of EP labs and are essential for determining the exact nature and source of arrhythmias. Future advances in these technologies are focused on enhancing imaging and mapping capabilities and improving environmental noise reduction, leading to more reliable diagnoses and accurate therapy delivery.

The development of more sensitive and accurate sensors and algorithms to filter out extraneous noise will allow a more accurate identification of the EGMs of interest and the precise localization of arrhythmias, reducing the risk of excess ablation while increasing efficacy. Furthermore, by reducing signal noise, procedures can be performed more swiftly and with greater confidence, reducing procedure duration and anesthesia times. Additionally, changes in signals post-ablation may be useful for the prediction of long-term ablation success and may be uniquely necessary in the era of pulsed field ablation.

Improvements in AF mapping by obtaining the highest fidelity source signals (eg, catheter–electrode combinations) for signal processing (eg, filtering, digitization, and noise elimination) are of the utmost importance to enable enhanced and automated interpretation of EGM recordings in the near future.^8^

With rising concerns regarding limiting the use of fluoroscopy and radiation exposure, the routine use of 3D electromagnetic mapping systems and ICE has become more prevalent. However, the costs and accessibility of these systems are still challenges for institutions globally, especially in the case of procedures with minimal risk or where fluoroscopy is equally effective. However, advancements in this area will be seen across multiple technologies.^9^

Although fluoroscopy, ultrasound, and electroanatomic maps are important guidance tools, these techniques do not provide the detailed anatomical information that can be obtained from high-resolution imaging. Future developments that allow advanced ICE, such as 4D ICE, which provides 3D images with the addition of a time dimension (4D) for real-time procedural guidance and superior spatial resolution, may allow for structural resolution usually seen with modalities that are not real time, such as computed tomography (CT) or magnetic resonance imaging (MRI). This would greatly enhance procedural workflows. These capabilities could, potentially, reduce procedure times and fluoroscopy use as well as reduce the equipment footprint in the EP lab. The most advanced ICE catheters available today provide enhanced definition of real-time anatomical structures and catheters integrated into an electroanatomic map, while reducing operator manipulation demands,^10^ as well as the need for preprocedural imaging.

### Signal recording

EP procedures were initially performed using only local and surface ECG signals and then ultimately progressed to the development of electroanatomic mapping. Despite the advances in 3D mapping, characterization of EGM signals remains crucial for EP procedures.^11^ The main advantage of signal recording is that it provides continuous high-resolution temporal data, allowing the measurement of real-time electrical activity in response to ablation procedures. A unified interface with multiple mapping systems is currently the central source of signals for various algorithms. Systems that facilitate integration with ML and AI will provide even more data to improve precision.

### Ablation modalities

Electroporation, intramural needle catheter ablation, and non-invasive radiotherapy ablation have been in development to help meet the challenge of treating arrhythmias.^12^ However, with so many emerging and unique technologies, the challenge for integration across recording and mapping systems must also be a focus for medical device companies.

### Combined tools, downsizing, and improved processing

Future developments and enhancements in EP lab technology will be related to a combination of different tools, downsizing, and improved processing capabilities, leading to more compact and powerful EP lab hardware. This transition will facilitate easier integration into the lab environment, reduce clutter, and improve accessibility. The shift toward more compact and integrated systems will also drive improvements in procedural efficiency and patient safety. Smaller, more versatile equipment will allow for quicker setup and transition between different types of procedures, reducing downtime and increasing the number of patients who can be treated. Moreover, with more streamlined workflows and fewer devices to manage, the potential for human error decreases, contributing to safer and more effective patient care.

### Connectivity and device integration

Future EP labs will feature seamless connectivity between various imaging, mapping, and ablative therapeutic modalities, achieving a “plug and play” state. Advanced device integration will simplify the inclusion of multiple inputs, making the system agnostic to the source while ensuring seamless operation. This will enhance the efficiency of procedures and improve patient outcomes. Achieving this level of integration will require robust and standardized communication protocols, enabling different devices and systems to interact effortlessly. Industry-wide collaboration and standardization efforts will be crucial in establishing these protocols and ensuring that new and existing technologies can coexist harmoniously.

## Data integration

### Centralized data storage and access

EP is inherently data-centric, with EGMs and other signals requiring extensive processing and filtering. Future advancements will involve the integration of cloud-based storage solutions that consolidate recording, mapping, and imaging data in a single, easily accessible location. This centralized approach will streamline data management and facilitate real-time access to comprehensive patient information on a large scale. An average EP procedure with recording, mapping, and imaging generates over 10 GB of raw data. Centralized data storage improves accessibility but also enhances data security and compliance with regulatory requirements. Cloud-based solutions can offer robust encryption and access control mechanisms, ensuring that sensitive patient data are protected from unauthorized access and breaches. Furthermore, centralized storage can facilitate data backup and disaster recovery, minimizing the risk of data loss due to hardware failures or other unforeseen events.

### Remote support and upgrades

Cloud-based remote support for equipment troubleshooting and software upgrades will become standard in EP labs. This capability will minimize downtime and ensure that the latest technologies and updates are readily available, enhancing overall lab efficiency and effectiveness.

### Data sharing for research and analysis

The ability to share de-identified data across hospitals and systems will be crucial for clinical research, quality improvement, outcome analysis, and economic evaluations. Data sharing will enable collaborative research efforts and support the development of ML and AI solutions, driving further innovations in EP.

The development of common tools and standardized formats is particularly challenging in the area of EP, a field that faces significant variety in experimental methodology, with a large number of data-acquisition systems, file formats that are often vendor-specific and undocumented, and a range of electrode catheter configurations. Currently, there is a lack of common organizational schemes or standards for accessing data. Thus, data exchange often requires substantial work to make the information accessible.^13^ The benefits of shared data will have an exponential effect on research and innovation, but to achieve this, data must be easier to clean and export and contain common data formats that align across multiple systems. This framework could enhance the functionality of hybrid operating room/EP lab models, integrating surgical procedures and imaging technologies, such as CT, MRI, and electrophysiological recording/mapping systems, into a unified platform with a more seamless experience for both operators and patients.

The combination of data from various sources, including imaging systems, electroanatomic mapping systems, and monitoring devices, also allows for a more comprehensive view of the patient’s condition. Integrated systems reduce the need for repeated measurements or imaging, as data from different modalities can be combined and utilized more effectively. Advanced integration allows for automation of routine tasks and enhanced procedural guidance, such as automated catheter navigation based on real-time data from multiple sources. Researchers and clinicians will be able to leverage larger datasets to identify trends and correlations that would be difficult to detect in smaller, siloed datasets. Moreover, standardized data formats will facilitate the development and deployment of AI and ML models, enabling these technologies to provide more accurate and actionable insights. Accurate data help in making precise adjustments during procedures and reduce the risks associated with human error. In addition, improved connectivity and integration processes optimize the use of imaging modalities, potentially reducing the reliance on fluoroscopy, thus lowering radiation exposure for both patients and staff.

## Workflow improvements/staff resource efficiencies

### Integrated consoles

Future EP labs will feature integrated consoles at the table side or in control rooms, consolidating fluoroscopy, intracardiac echo, mapping, and table functionality. This integration will streamline workflows, reduce the need for multiple devices, enhance procedural efficiency, reduce equipment and operational costs, and drive efficient preprocedural setup and planning. The improvements in procedural scheduling and resource allocation, together with automated patient data integration, will allow for timely and well-prepared interventions. Advanced scheduling algorithms can optimize the use of resources, including personnel and equipment, reducing downtime and ensuring that procedures are conducted as efficiently as possible. Additionally, automated patient data integration can reduce the time and effort required to prepare for each procedure, allowing clinicians to focus more on patient care.

### Enhanced charting and report generation

Automated charting and report generation systems, incorporating voice recognition and shortcut functionalities, will improve documentation efficiency for physicians, nurses, and technologists.

While speech recognition for health care use has faced challenges, recently, it has been described that speech recognition for documentation during surgical procedures is more accurate, efficient than other methods, and improves the quality of documentation as well as increases the uptake of digital transformation in facilities.^14^

EP systems will integrate and speak the language of the electronic medical record, so unique data and images will be visualized within the record without having to access multiple systems within the EP lab. All repositories should be developed to be more easily accessed and readable.

### Remote, real-time communication for workflow automation and staff flexibility

While workflow automations will streamline many processes, the importance of skilled EP nurses, radiology technologists, and clinical mapping specialists will remain paramount. These professionals will benefit from increased flexibility, allowing them to meet rising case demands, address geographical challenges, and continuously grow their competencies. The integration of new technologies should be designed to augment rather than replace these critical roles.

Real-time communication and collaboration tools will be integral to future EP labs. Advanced communication systems will allow multidisciplinary teams to coordinate efforts seamlessly, improving patient care and procedural efficiency. Secure messaging platforms, video conferencing, and collaborative software will facilitate effective communication among team members, regardless of their physical location.

Real-time communication systems will be particularly beneficial in complex cases that require input from multiple specialists. For example, during a challenging ablation procedure, an electrophysiologist might need to consult with a cardiologist, a radiologist, or another expert electrophysiologist. With advanced communication tools, these consultations can happen in real time, with specialists providing input and guidance from remote locations. This collaborative approach can enhance decision-making and improve patient outcomes. Additionally, as the demand for expert mapping specialists outpaces the supply, remote applications will be critical to allow support by one specialist for several EP labs without having to physically relocate for each procedure.

### Education/expert guidance in a remote environment that paves the way for global access

The future of EP labs must address global disparities in health care access. Efforts to make advanced EP technologies more affordable and accessible in low-resource settings will be critical.

Improving accessibility in low-resource settings will require a multifaceted approach. On one hand, developing cost-effective equipment that can perform essential functions without compromising quality will be crucial. Additionally, providing remote support through telemedicine and remote monitoring can extend the reach of EP services, allowing clinicians in low-resource settings to access expertise and guidance from specialists in other locations. Establishing training programs to build local expertise will also be fundamental to ensure that clinicians in low-resource settings have the skills and knowledge to provide high-quality care.

The incorporation of virtual reality (VR) tools is revolutionizing education and expert guidance in EP labs. These tools will enable providers in less experienced centers to receive real-time expert support, expanding access to EP services in underserved regions. VR technologies will also facilitate immersive training experiences, allowing trainees to engage in realistic, high-stakes scenarios safely and consistently.^15^

There are many regions of the world that are without basic cardiac EP services and infrastructure.^16^ Remote connectivity will enable global training and education, breaking down geographical barriers. Providing care that conforms to guidelines is of pivotal importance to optimize patient outcomes, but adherence to the guidelines is often suboptimal. The availability of interactive clinical practice guidelines through an application can help ensure that treatment conforms to guidelines.^17^ Through virtual platforms, trainees and physicians can experience the lab environment and interact with all inputs as if they were physically present. This approach will standardize training and ensure high-quality education across diverse settings.

## Conclusion

The future of EP labs lies in integrating advanced technologies, optimizing hardware and infrastructure, enhancing data integration and workflow efficiencies, and prioritizing education and global accessibility. By addressing these key areas, EP labs that are more flexible, efficient, and accessible can be created, ultimately improving patient care and outcomes. As the medical and research communities continue to innovate and collaborate, the future of EP holds immense promise for transforming cardiac care and advancing the field.

**Figure 1:**
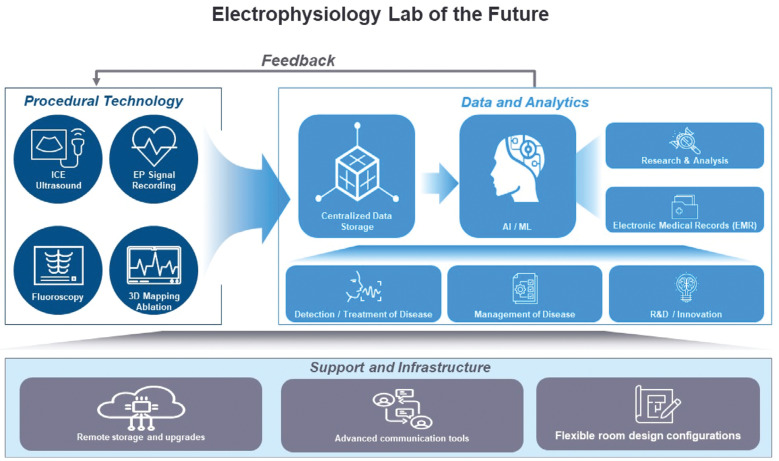
Central illustration—key elements of the electrophysiology lab of the future. *Abbreviation:* ICE, intracardiac echocardiography.

**Figure 2:**
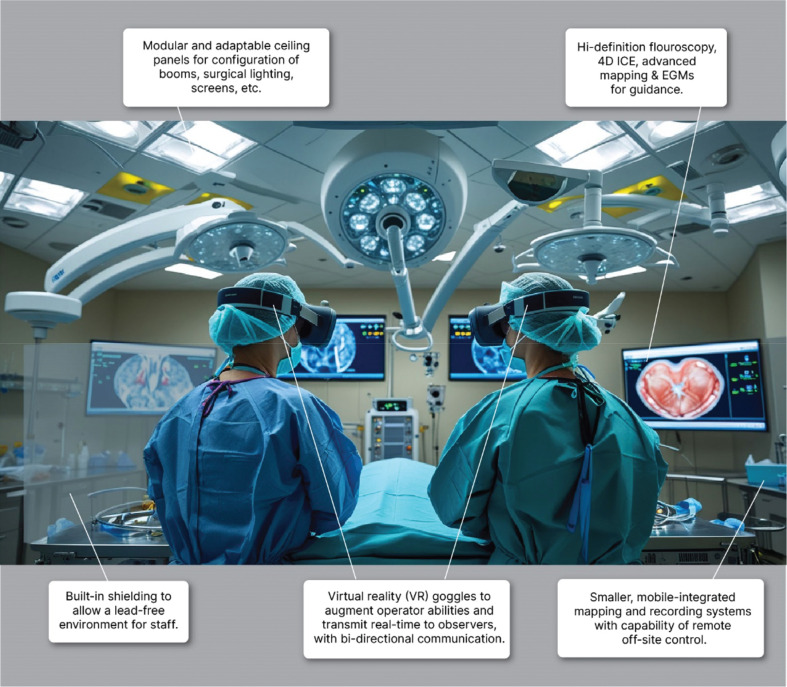
Visual display with design elements of a proposed electrophysiology lab of the future. *Abbreviations:* 4D ICE, four-dimensional intracardiac echocardiography; EGM, electrogram.

Designing and building an EP lab today requires a forward-thinking approach that anticipates future advancements and addresses current challenges. Embracing cutting-edge technologies, optimizing workflows, and fostering a culture of continuous improvement and collaboration will allow the creation of EP labs that are well equipped and that meet the needs of patients and clinicians now and in the future. The journey toward creating the ideal EP lab is ongoing, but with a clear vision and a commitment to innovation, significant progress can be made with a long-lasting impact on the field of EP **(Figures 1 and 2)**. Collaboration and partnerships are key drivers of innovation and patient care improvement in EP; therefore, it is fundamental to keep identifying and discussing the needs of the EP labs of the future.

Importantly, integration challenges are not just technical but also regulatory and procedural. Different devices and systems may need to comply with varying standards and regulations while ensuring interoperability. Additionally, procedural protocols might need to be adapted to leverage the full potential of integrated systems, requiring training and adjustments in workflow. With the integration of advanced data systems, ensuring the privacy and security of patient data is paramount. Compliance with regulations such as the Health Insurance Portability and Accountability Act (HIPAA) in the United States, or the General Data Protection Regulation (GDPR) in Europe, is essential. This involves implementing robust data-protection measures, conducting regular security audits, and providing staff with training on data privacy practices. This will require medical device companies to drive these advancements across their organization.

The EP lab of the future is coming and allows a never-before opportunity for the coalescence of multiple types of technological advancements, human factors input, design, and customization, to create scientific platforms that completely revolutionize cardiac EP for the benefit of patients around the world.
